# Evaluating interactions of patients with large language models for medical information

**DOI:** 10.1111/bju.16676

**Published:** 2025-02-18

**Authors:** Nicolas Carl, Sarah Haggenmüller, Christoph Wies, Lisa Nguyen, Jana Theres Winterstein, Martin Joachim Hetz, Maurin Helen Mangold, Friedrich Otto Hartung, Britta Grüne, Tim Holland‐Letz, Maurice Stephan Michel, Titus Josef Brinker, Frederik Wessels

**Affiliations:** ^1^ Department of Urology, University Medical Center Mannheim Ruprecht‐Karls University of Heidelberg Mannheim Germany; ^2^ Medical Faculty Ruprecht‐Karls University of Heidelberg Mannheim Germany; ^3^ Medical Faculty Mannheim Ruprecht‐Karls University of Heidelberg Mannheim Germany; ^4^ Division of Digital Prevention, Diagnostics and Therapy Guidance German Cancer Research Center (DKFZ) Heidelberg Germany; ^5^ Department of Biostatistics German Cancer Research Center (DKFZ) Heidelberg Germany

**Keywords:** clinical trial, artificial intelligence, implementation science, large language models, patient interaction

## Abstract

**Objectives:**

To explore the interaction of real‐world patients with a chatbot in a clinical setting, investigating key aspects of medical information provided by large language models (LLMs).

**Patients and methods:**

The study enrolled 300 patients seeking urological counselling between February and July 2024. First, participants voluntarily conversed with a Generative Pre‐trained Transformer 4 (GPT‐4) powered chatbot to ask questions related to their medical situation. In the following survey, patients rated the perceived utility, completeness, and understandability of the information provided during the simulated conversation as well as user‐friendliness. Finally, patients were asked which, in their experience, best answered their questions: LLMs, urologists, or search engines.

**Results:**

A total of 292 patients completed the study. The majority of patients perceived the chatbot as providing useful, complete, and understandable information, as well as being user‐friendly. However, the ability of human urologists to answer medical questions in an understandable way was rated higher than of LLMs. Interestingly, 53% of participants rated the question‐answering ability of LLMs higher than search engines. Age was not associated with preferences. Limitations include social desirability and sampling biases.

**Discussion:**

This study highlights the potential of LLMs to enhance patient education and communication in clinical settings, with patients valuing their user‐friendliness and comprehensiveness for medical information. By addressing preliminary questions, LLMs could potentially relieve time constraints on healthcare providers, enabling medical personnel to focus on complex inquiries and patient care.

**Abbreviations**
AI: artificial intelligence
GPT‐4: Generative Pre‐trained Transformer 4
IPaLLM: Interaction of Patients with Large Language Models
LLM: large language model
medQA: medical question‐answering
OR: odds ratio

## Introduction

Large language models (LLMs) are advanced tools capable of processing human natural language, excelling in ‘zero‐shot’ tasks such as data extraction, information structuring, medical note summarisation, enhancing the readability of medical reports and medical question‐answering (medQA) [1, 2, 3, 4, 5]. These powerful capabilities stem from their embedded medical knowledge, which has captivated researchers and industry professionals alike [6, 7]. Global surveys indicate that 60–80% of clinicians and researchers utilize LLMs for both clinical and academic purposes [8, 9]. Specifically, half of urologists surveyed believe that LLMs could enhance patient education and support clinical decision‐making [9].

An expanding body of research demonstrates that LLMs can accurately respond to urological questions with a high degree of accuracy [10, 11, 12, 13, 14, 15, 16]. Beyond accuracy, LLMs offer a dynamic, personalised approach, allowing users to obtain precise answers to medical questions without the need to sift through lengthy articles. Their user‐friendly interfaces and ability to provide tailored, accessible information have positioned LLMs as promising tools for medical education [17, 18]. Consequently, it seems feasible that, in the near future, LLMs could reduce barriers in patient education, potentially supplementing or even replacing search engines as primary sources of medical information [19, 20].

Despite surveys suggesting that clinicians are integrating artificial intelligence (AI) tools into their practices, real‐world urological patients have limited user experience with AI technologies [21, 22]. To date, patients have largely been left out of this technological evolution and data on how patients interact with AI for medical information‐seeking, particularly LLMs, remains limited. To investigate how patients might independently utilise a chatbot like ChatGPT to learn about medical conditions, a study allowing open interaction with the chatbot and evaluating users’ perceptions of their understanding and the information's usefulness could yield meaningful insights [23]. To the best of our knowledge, no such study has been conducted in the field of urology.

To bridge this gap in literature, our study explored the perceived utility and completeness, understandability, and user‐friendliness of LLMs in a real‐world urological patient sample. Given an anticipated lack of prior AI experience among patients, we designed a prospective clinical trial that introduced patients to LLMs in a controlled setting. This approach was intended to minimise bias by providing hands‐on experience, enabling a more realistic assessment of patient interactions with LLMs for medQA. Additionally, to assess patient preferences for obtaining medical information, we evaluated their opinions on LLMs compared to the quality of information provided by search engines or by consultations with human urologists.

## Patients and Methods

This work is part of the clinical study ‘Interaction of Patients with Large Language Models’ (IPaLLM) and shares a unified study design and methodology with part one entitled ‘Comparing Patient's Confidence in Clinical Capabilities in Urology: Large Language Models Versus Urologists’, which has been recently published [23].

### Reporting Standards and Ethics Statement

We adhered to the Transparent Reporting of a multivariable prediction model for Individual Prognosis Or Diagnosis (TRIPOD)‐LLM statement as a reporting guideline, with a corresponding checklist provided in Data S1 [24]. The presented results are part of a prospective the IPaLLM clinical trial, officially registered with the German Clinical Trial Registry (Deutsches Register Klinischer Studien identifier [DRKS‐ID]: 00034906). This study was performed in line with the principles of the Declaration of Helsinki and was approved by the institutional ethics review board of the Medical Faculty Mannheim, Ruprecht‐Karls University of Heidelberg (proposal number: 2023‐687).

### Patient Enrolment

Patients attending counselling regarding elective urological surgery at the University Medical Center Mannheim were prospectively enrolled on‐site. Patients presented to our clinic for elective surgical preparation for a variety of procedures, including uro‐oncological surgery (e.g., surgeries for prostate, kidney, testicular, and bladder cancer), endourological treatments (e.g., BPH and urolithiasis), and reconstructive or transgender surgeries. To be eligible, participants had to be aged ≥18 years, with individuals having cognitive impairments or psychiatric conditions excluded from the study. All participants provided informed consent.

### Study Design and Procedure

**Fig. 1:**
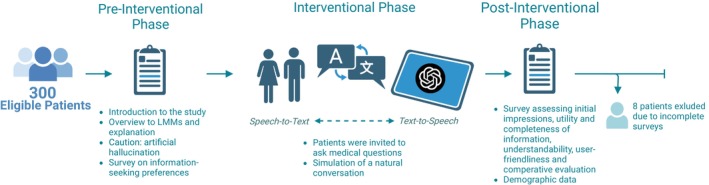
The IPaLLM study design and procedure. Participants received an introduction to LLMs and completed a pre‐interventional survey assessing their information‐seeking preferences and AI affinity. During the interventional phase, they interacted with a GPT‐4 powered chatbot in a controlled setting, posing medical questions relevant to their current clinical situations. In the post‐interventional phase, participants completed a survey evaluating their initial impressions of the chatbot, including its comprehension, user‐friendliness, and their preferences compared to search engines and consultations with urologists. This figure is adapted from part I of the IPaLLM study [23].

This work presents results from the larger IPaLLM trial, which investigated patient perspectives on the clinical capabilities of LLMs [23]. While the broader IPaLLM study examined clinical capabilities of LLMs, this part of the trial focuses specifically on how patients perceive the quality of the medical information provided by Generative Pre‐trained Transformer 4 (GPT‐4; OpenAI Inc., San Francisco, CA, USA). The on‐site procedure consisted of three phases: a pre‐interventional phase to introduce the study, an interventional phase featuring a guided chatbot conversation, and a post‐interventional survey to assess patients’ perspectives on the use of AI in clinical workflows (see Fig. 1). The study design aimed to lower barriers for older patients, a significant demographic in urology, by offering a guided introduction to LLMs within a controlled setting.

#### Pre‐Interventional Phase

Participants received an explanation of the experiment, an overview of LLMs, and the ‘prompt‐answer’ principle, along with a warning about potential artificial hallucinations (i.e., incorrect but seemingly accurate information). This was supplemented by a detailed information sheet. Participants subsequently completed a preliminary survey to evaluate their preferences for obtaining medical information, foundational knowledge, and AI affinity.

#### Interventional Phase

Participants interacted with a GPT‐4 powered chatbot (OpenAI) via a browser application on a tablet, using speech‐to‐text for input and text‐to‐speech for responses, simulating a natural conversation. Participants were encouraged to pose medical questions relevant to their ongoing clinical conditions or planned procedures. The chatbot was preprompted with keywords tailored to each clinical context (e.g., prostate biopsy or nephrolithiasis). Participants were guided to ask questions in a natural manner, similar to how they would interact with a physician or search for information on‐line. The study took place in a controlled setting, with personnel on site to address any inaccuracies in the responses of the chatbot.

#### Post‐Interventional Phase

After interacting with the chatbot, participants completed a post‐interventional survey and demographic questionnaire anonymously. To ensure the anonymity of survey responses, medical supervisors were not present during this step. The entire process lasted ~30 min for each participant. Following this, the tablets were retrieved, and study personnel addressed any outstanding questions or instances of artificial hallucinations to resolve potential inaccuracies.

### Questionnaire Development

We initially reviewed existing literature, followed by six semi‐structured expert interviews conducted with three patients and three board‐certified urologists (F.W., F.O.H., B.G.) to gain comprehensive insights. Based on these findings, the survey questionnaire and intervention were developed and subsequently reviewed by the interviewed urologists and an AI specialist (S.H.). To validate clarity and consistency, the finalised survey and chatbot intervention were tested by 20 medical students without prior expertise in AI.

The survey was designed to assess patients’ perspectives on LLMs, exploratively deployed as information tools. The first subsection assessed participants’ initial impressions, including whether they enjoyed the interaction, felt their questions were understood, and perceived the chatbot as competent. The second subsection evaluated the perceived utility and completeness of the information provided, using separate items for each category. In the third subsection, understandability of the responses was assessed with three items, focusing on provided content and explanations as well as technical language used. The fourth subsection addressed user‐friendliness. In the fifth subsection, comparative items were introduced to determine whether participants felt that GPT‐4 answered questions better than human urologists or search engines. Agreement for items was captured using a Likert scale ranging from 1 (‘strongly disagree’) to 5 (‘strongly agree’). The survey was conducted in German. A translated English version was made available for reporting purposes (Table S1).

### Statistical Analysis

To examine associations between question outcomes and participant demographics, an ordinal logistic regression analysis was conducted. Odds ratios (ORs) were calculated by applying the exponential function on β coefficients. The 95% CI (using the modified Wald method [25]) where derived from the coefficients CIs in the same way, and *P* values denoting whether ORs differ from 1 were reported for the ORs included into the explorative regression analysis: age, affinity for AI, prior use of ChatGPT or other LLM, confidence in online sources, and education level. The overall significance of the parameters of the model was assessed using *P* values, with a significance threshold of 5% for all hypothesis testing. The distributions of questionnaire responses were visualised using Likert plots. Data analysis was performed using R (version 4.1.2; R Foundation for Statistical Computing, Vienna, Austria). As providing demographic data was voluntary, discrepancies from 100% occur due to missing entries, which were excluded from the analysis. Additionally, values were rounded or truncated to obtain natural numbers for subsequent analysis. For better readability, we report ‘agree’ and ‘strongly agree’ as positive feedback, while ‘disagree’ and ‘strongly disagree’ are categorised as negative feedback.

## Results

### Study Sample Represents a Real‐Life Distributed Urological Cohort with Limited AI Experience

Of the 300 patients prospectively enrolled, 292 completed the study, resulting in a response rate of 97.3%. The cohort consisted of 212 male and 71 female participants, with a median (range) age of 64 (18–96) years. Comprehensive demographic details are presented in Table S2. In part one of the IPaLLM study, we gathered data on the technology use habits of our cohort, revealing that the majority of participants (82%, 95% CI 73.3–88.3%) used Google for medical information, with 48% (95% CI 38.5–57.7%) accessing the internet daily for this purpose. AI affinity was limited, with only 8% (95% CI 4.1–15.0%) of participants having prior experience with LLMs for medical queries. Younger adults showed a higher tendency to use LLMs compared to older participants [23].

### Positive Reception: Perceived Initial Impressions, Utility, Completeness, Understandability, and User‐Friendliness

**Fig. 2:**
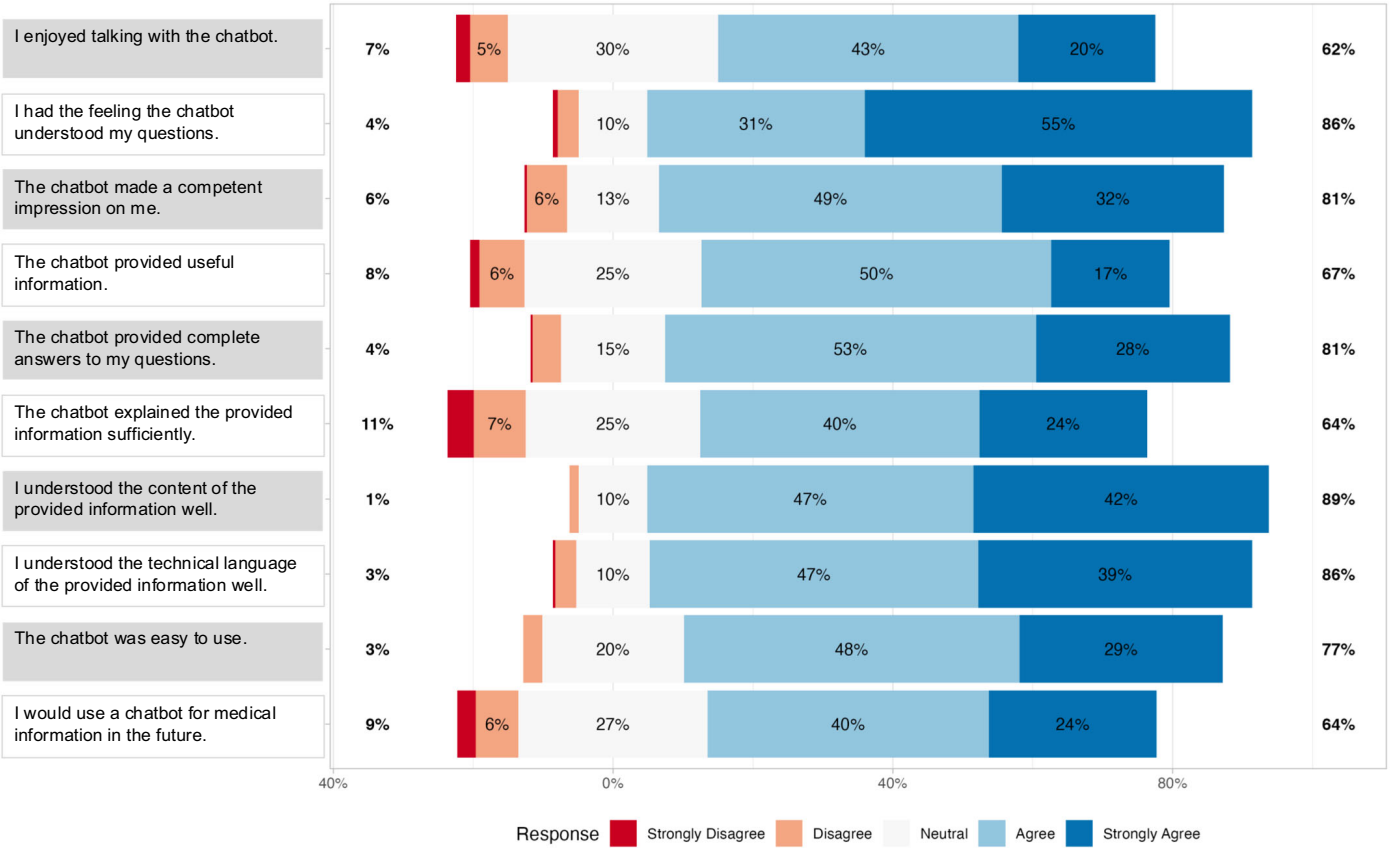
Likert plot displaying post‐interventional survey results initial impressions, perceived utility, completeness, and understandability of the chatbot responses as well as user‐friendliness. Each row represents one survey item, with results shown as percentages for each level of a 5‐point Likert scale. The levels range from ‘strongly disagree’ to ‘strongly agree’, with percentages on the far left and right indicating the combined totals of ‘strongly ‘disagree/disagree’ and ‘agree/strongly agree’ respectively.

Initially, we assessed patients’ initial impressions, including their perceived utility, completeness, understandability of the provided chatbot responses, and overall user‐friendliness, to gauge general sentiment on the interaction with the chatbot (Fig. 2).

Patients reported positive feedback on the initial impressions of the chatbot conversation, with 62% (95% CI 56.44–67.41%) stating that they enjoyed interacting with it and 86% (95% CI 82.56–90.29%) indicating that the chatbot understood their questions. Additionally, 81% (95% CI 75.99–84.94%) of patients felt that the chatbot made a competent impression. Furthermore, the majority of 67% (95% CI 61.24–71.88%) rated the provided information as useful, while 81% (95% CI 75.99–84.94%) of patients reported that the chatbot completely answered their questions. Concerning the understandability of the delivered information, 64% (95% CI 58.49–69.32%) of patients reported sufficient explanations to the questions and found both the medical content and technical language to be understandable, with 89% (95% CI 85.18–92.31%) and 86% (95% CI 82.19–90.0%) providing positive feedback, respectively. Moreover, 77% (95% CI 72.42–81.89%) reported that the chatbot was easy to use. Overall, patients reported positive first impressions and positively rated utility, completeness, understandability of chatbot responses as well as user‐friendliness. Finally, the majority of 64% (95% CI 58.49–69.32%) of patients showed interest in using a chatbot for medical information in the future.

### Patients Prefer LLMs over Search Engines, but Urologists Remain the Go‐to for Medical Advice

**Fig. 3:**
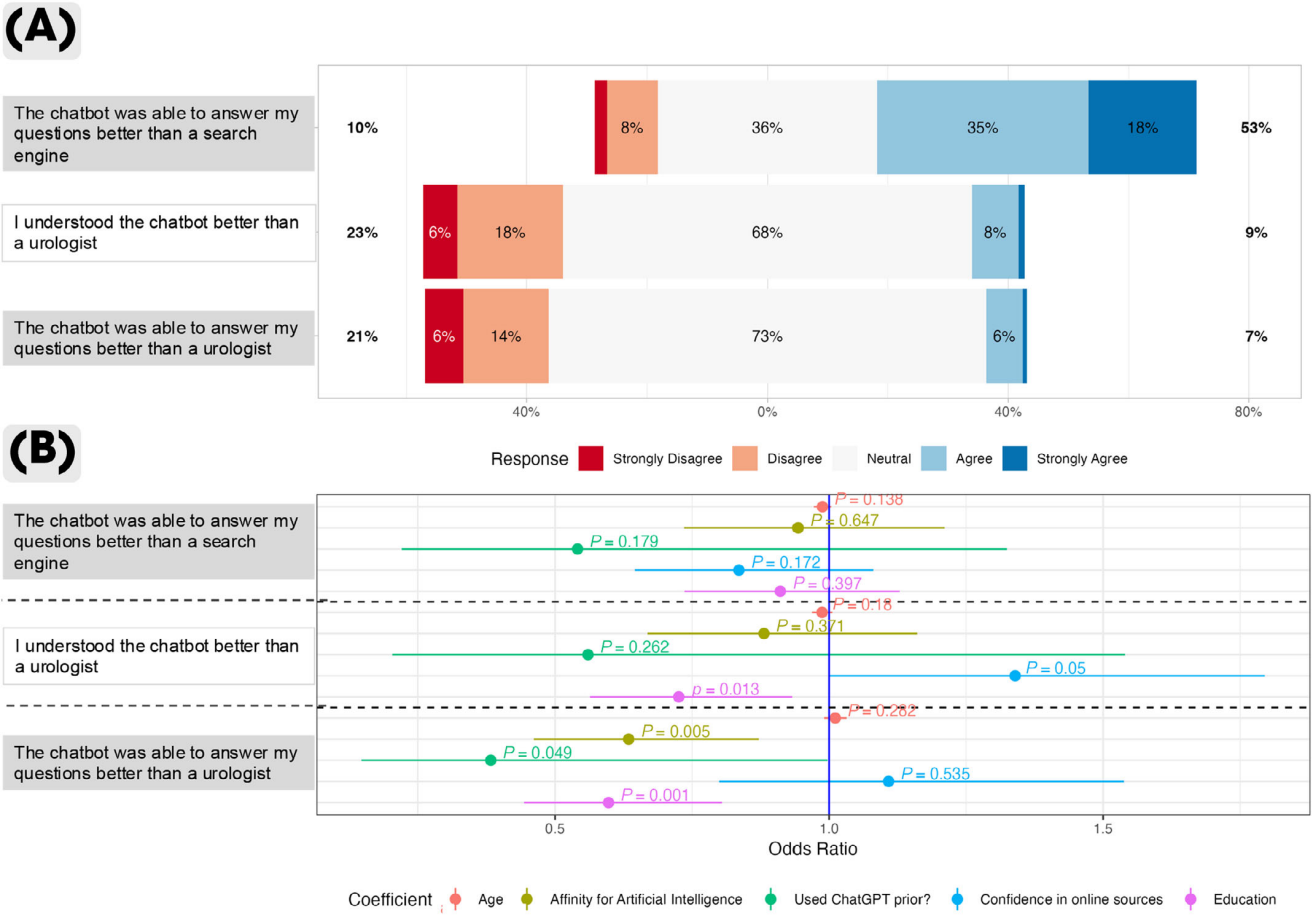
(**A**) Likert plot displaying post‐interventional survey results on patient preferences. (**B**) Forest plot displaying results of a logistic ordinal regression on patient preferences. (**A**) Each row represents one survey item, with results shown as percentages for each level of a 5‐point Likert scale. The levels range from ‘strongly disagree’ to ‘strongly agree,’ with percentages on the far left and right indicating the combined totals of ‘strongly disagree/disagree’ and ‘agree/strongly agree,’ respectively. (**B**) Forest plot displays the results of an ordinal logistic regression model. The regression analysis was conducted to evaluate the probability of patients agreeing with the statement. The direction of included factors were higher age, greater affinity for AI, prior use of ChatGPT, higher confidence in on‐line sources, and higher levels of education. Each row represents one survey item, the vertical line around zero represents the no‐effect line (blue line), Odds are displayed on the *x*‐axis as single dots with the corresponding horizontal line representing the 95% CI. A factor was considered significant when the 95% CI did not overlap with the no‐effect line, i.e., if the value 1 was not included in the 95% CI.

Next, we explored whether patients rated the ability of GPT‐4 to answer their questions more favourably than search engines or human urologists (Fig. 3A). Additionally, we conducted an explorative ordinal logistic regression analysis to identify patient factors potentially associated with these preferences (Fig. 3B).

A majority of 53% reported that the chatbot answered their questions better than search engines. However, more patients disagreed when asked if the chatbot was easier to understand or provided better answers than a urologist, with 23% and 21% of the respondents providing negative feedback, respectively. Notably, ~70% of respondents selected a neutral option, suggesting uncertainty about comparing LLMs with urologists.

The regression analysis revealed that confidence in online information sources was statistically significantly associated with the perceived understandability of the chatbot (OR 1.34, 95% CI 1.00–1.80; *P* = 0.05). Conversely, higher educational levels were associated with a higher likelihood of patients disagreeing that GPT‐4 provides better (OR 0.73, 95% CI 0.56–0.93; *P* = 0.013) and more understandable answers (OR 0.60, 95% CI 0.44–0.80; *P* = 0.001) compared to a human urologist. Interestingly, patients with a greater affinity for AI and prior chatbot experience were more likely to disagree with the statement that GPT‐4 could answer questions better than a human urologist. Detailed results are presented in Fig. 3B. Overall, our findings indicate that patients rated urologists higher than GPT‐4 for both the ability and understandability of medQA, while GPT‐4 was rated more favourably than search engines for medQA. Additionally, the regression analysis suggests a trend linked to educational levels but does not support an association between age and perceived understandability and quality of the LLM compared to search engines or human urologists.

## Discussion

This study examined the integration of LLMs within a clinical setting, allowing patients hands‐on experience to assess their perspectives on the ability of GPT‐4 used as a speech‐to‐speech chatbot to provide medical information. Our findings indicate that patients report positive initial impressions, with favourable ratings for perceived utility, completeness, understandability, and user‐friendliness of chatbot responses. However, human urologists were rated higher than GPT‐4 in both quality and understandability. Interestingly, most patients preferred GPT‐4 over search engines and expressed interest in using LLMs in the future. Notably, patient preferences were not affected by age, suggesting that LLMs are accessible and user‐friendly across diverse groups. In summary, our results provide preliminary evidence that urological patients are interested in using AI for medical information. The practical applications of these findings indicate that LLMs could be integrated for preliminary medical questions, but human expertise remains crucial, particularly for complex inquiries.

Search engines (e.g., Google) are currently the primary source of medical information for most patients, with only a minority, particularly younger individuals, having used chatbots for medical advice [19, 23]. However, LLM‐based chatbots are increasingly discussed as tools that could supplement or even replace search engines [20]. Our study supports this hypothesis, with 53% of participants agreeing that LLMs provide better answers than search engines, highlighting the potential of LLMs to reshape how patients access medical information in the future.

Our study results demonstrate that most patients appreciate the user‐friendliness and quality of information delivered by a chatbot, which aligns with prior *in silico* research demonstrating high accuracy in medQA [5, 13]. LLMs can bridge gaps for patients who struggle to navigate cluttered, complex medical literature on‐line by simplifying medical language and enhancing readability [10, 13, 26]. Also, research shows that patients who conduct pre‐consultation research tend to have a better understanding after meeting with their urologist [27]. The latest versions of LLMs like Perplexity AI and GPT‐4 now integrate live internet queries, allowing them to retrieve real‐time information, such as contact details for local urologists. This capability enables LLMs to provide both educational content and practical information, positioning them as a comprehensive alternative to traditional search engines for pre‐consultation research.

In this study, patients initially responded positively to the LLM‐powered chatbot, appreciating its utility, completeness, understandability, and user‐friendliness. However, this positive reception may reflect only the technical strengths of LLMs, as more patients ultimately favoured urologists for understandability and answer quality and most remained uncertain. The regression analysis revealed that more educated patients and those with prior chatbot experience were more sceptical of LLMs, suggesting that more informed patients may view LLMs critically. Prior exposure to LLMs may increase scepticism, possibly due to recognising their limitations of artificial hallucinations, which remains a key limitation of LLM‐based chatbots. In our study, any inaccuracies or outdated information provided by the LLM were corrected by study personnel. This clinical yet controlled setting differs from real‐world applications, where artificial hallucinations could lead to potentially harmful misinformation.

There is a noticeable lack of studies evaluating the effectiveness of AI solutions in real‐world clinical settings [28, 29]. Moving forward, it is crucial to understand how AI can be effectively implemented in clinical workflows while ensuring patient safety. IPaLLM aims to bridge this gap by providing evidence and facilitating the integration of AI within a controlled clinical environment to assess its impact on and perspectives from urological patients.

This study is subject to limitations, particularly due to potential social desirability bias, which may have arisen from the direct questioning approach used. To mitigate this, we are utilising the findings to develop a follow‐up study that will implement indirect questioning techniques to minimise this bias. Additionally, there may be sampling bias as the cohort primarily consisted of older male participants. Nevertheless, this demographic closely aligns with the typical patient population affected by urological conditions in clinical practice. Moreover, the controlled environment and standardised methodologies of the study enhance its overall validity and reliability. Furthermore, this study may be subject to language bias, as the investigated LLM was employed in German to align with the German patient cohort. As the training data for most current LLMs is dominated by the English language, response quality for non‐English languages, including German, may be reduced [30]. However, language‐based performance differences in medQA specifically requires investigation in future studies. To our knowledge, no validated survey tool exists specifically for patients interacting with AI, leaving no established orientation for comprehensive validity testing. Therefore, we designed this study in an exploratory manner to lay the groundwork for future research. No formal power calculation was conducted, as this exploratory study lacked reference points being the first of its kind known to us.

## Conclusion

This study highlights the potential of LLMs to enhance patient education and communication in clinical settings. Patients appreciated the user‐friendliness and comprehensiveness of AI‐generated information on their individual urological questions, showing a preference for LLMs over search engines for accessing medical information, although they preferred human urologists for medical advice. The absence of age‐related differences suggests that LLMs are accessible across diverse groups, including elderly patients. Utilising LLMs as educational tools for preliminary medical questions, could therefore help relieve time constraints on healthcare providers, allowing urologists to dedicate more attention for complex inquiries, shared decision‐making and patient care.

## Author Contributions

Nicolas Carl developed the study concept, designed the methodology, conducted the investigation, collected data, performed the statistical analysis, validated the findings, authored the original draft, and created visual representations. Sarah Haggenmüller supported the conceptualisation, contributed to the validation process, participated in the review and editing of the manuscript and supervised the project team. Christoph Wies contributed to the study by conducting and validating statistical analysis, creating illustrations, review and editing of the manuscript. Lisa Nguyen contributed to the study with data collection. Jana Theres Winterstein contributed by creating visual representations and review of the manuscript. Martin Joachim Hetz contributed by creating visual representations and review of the manuscript. Maurin Helen Mangold contributed by validating the questionnaire and review of the manuscript. Friedrich Otto Hartung contributed by validating the questionnaire and review of the manuscript. Britta Grüne contributed by validating the questionnaire and review of the manuscript. Tim Holland‐Letz contributed to the study by validating the statistical analysis, review and editing of the manuscript. Maurice Stephan Michel provided resources and participated in the review of the manuscript. Titus Josef Brinker supported conceptualisation, provided resources, reviewed and edited the manuscript, supervised the project team, administered the project, and acquired funding. Frederik Wessels led the conceptualisation together with the corresponding author, supervised design of methodology, reviewed and edited the manuscript and supervised the project.

## Disclosure of Interests

The authors declare the following financial interests/personal relationships which may be considered as potential competing interests: Frederik Wessels would like to disclose that he advises Astellas, AstraZeneca, Janssen and Adon Health outside of the submitted work. The other authors have no conflicts of interest to declare. Titus Josef Brinker would like to disclose that he is the owner of Smart Health Heidelberg GmbH (Handschuhsheimer Landstr. 9/1, 69120 Heidelberg, Germany; www.smarthealth.de), outside of the submitted work.

## Funding

The research is funded by the Ministry for Social Affairs, Health and Integration, Baden Württemberg, Germany. The funder had no role in study design, data collection and analysis, decision to publish, or preparation of the manuscript.

## Consent to Participate

Informed consent was obtained from all individual participants included in the study.

## Supporting information


**Data S1.** The TRIPOD‐LLM statement: a targeted guideline for reporting large language models use.

**Table S1.** Post‐interventional survey.
**Table S2.** Demographic data and medical information‐seeking preferences.
**Table S3.** Descriptive Table with the reason of admittance.
**Table S4.** Results of a ordinal logistic regression model.
**Figure S1.** Histogram detailing reason of admittance.

## Data Availability

Due to privacy concerns, the data have not been made publicly available. However, all data collected or analysed during this study are available from the corresponding author upon reasonable request. Please contact Nicolas Carl at nicolas.carl@umm.de for access to the data. The code for statistical analysis is provided separately in Data S1.

## References

[1] Kather JN , Ferber D , Wiest IC , Gilbert S , Truhn D . Large language models could make natural language again the universal interface of healthcare. Nat Med 2024; 30: 2708–2710 39179856 10.1038/s41591-024-03199-w

[2] Brown TB , Mann B , Ryder N et al. Language Models are Few‐Shot Learners [Internet]. *arXiv* 2020. http://arxiv.org/abs/2005.14165

[3] Perez‐Lopez R , Ghaffari Laleh N , Mahmood F , Kather JN . A guide to artificial intelligence for cancer researchers. Nat Rev Cancer 2024; 24: 427–441 38755439 10.1038/s41568-024-00694-7

[4] Iannantuono GM , Bracken‐Clarke D , Floudas CS , Roselli M , Gulley JL , Karzai F . Applications of large language models in cancer care: current evidence and future perspectives. Front Oncol 2023; 13: 1268915 37731643 10.3389/fonc.2023.1268915 PMC10507617

[5] Ayers JW , Poliak A , Dredze M et al. Comparing physician and artificial intelligence chatbot responses to patient questions posted to a public social media forum. JAMA Intern Med 2023; 183: 589–596 37115527 10.1001/jamainternmed.2023.1838 PMC10148230

[6] Singhal K , Azizi S , Tu T et al. Large language models encode clinical knowledge. Nature 2023; 620: 172–180 37438534 10.1038/s41586-023-06291-2 PMC10396962

[7] Lee P , Bubeck S , Petro J . Benefits, limits, and risks of GPT‐4 as an AI chatbot for medicine. N Engl J Med 2023; 388: 1233–1239 36988602 10.1056/NEJMsr2214184

[8] Owens B . How nature readers are using ChatGPT. Nature 2023; 615: 20 36807343 10.1038/d41586-023-00500-8

[9] Eppler M , Ganjavi C , Ramacciotti LS et al. Awareness and use of ChatGPT and large language models: a prospective cross‐sectional global survey in urology. Eur Urol 2023; 85: 146–153 37926642 10.1016/j.eururo.2023.10.014

[10] Davis R , Eppler M , Ayo‐Ajibola O et al. Evaluating the effectiveness of artificial intelligence‐powered large language models application in disseminating appropriate and readable health information in urology. J Urol 2023; 210: 688–694 37428117 10.1097/JU.0000000000003615

[11] Hetz MJ , Carl N , Wies C et al. Superhuman performance on urology board questions using an explainable language model enhanced with European Association of Urology guidelines. ESMO Real World Data Digit Oncol 2024; 6: 100078 10.1016/j.esmorw.2024.100078 PMC12836625 41646097

[12] Kollitsch L , Eredics K , Marszalek M et al. How does artificial intelligence master urological board examinations? A comparative analysis of different large language models' accuracy and reliability in the 2022 in‐service assessment of the European Board of Urology. World J Urol 2024; 42: 20 38197996 10.1007/s00345-023-04749-6

[13] Hershenhouse JS , Mokhtar D , Eppler MB et al. Accuracy, readability, and understandability of large language models for prostate cancer information to the public. Prostate Cancer Prostatic Dis 2024. 1–6 38744934 10.1038/s41391-024-00826-y PMC12106072

[14] Caglar U , Yildiz O , Meric A et al. Evaluating the performance of ChatGPT in answering questions related to pediatric urology. J Pediatr Urol 2024; 20(1): 26.e1–26.e5 10.1016/j.jpurol.2023.08.003 37596194

[15] Coskun B , Ocakoglu G , Yetemen M , Kaygisiz O . Can ChatGPT, an artificial intelligence language model, provide accurate and high‐quality patient information on prostate cancer? Urology 2023; 180: 35–58 37406864 10.1016/j.urology.2023.05.040

[16] Zhou Z , Wang X , Li X , Liao L . Is ChatGPT an evidence‐based doctor? Eur Urol 2023; 84: 355–356 37061445 10.1016/j.eururo.2023.03.037

[17] Clusmann J , Kolbinger FR , Muti HS et al. The future landscape of large language models in medicine. Commun Med 2023; 3: 141 37816837 10.1038/s43856-023-00370-1 PMC10564921

[18] Safranek CW , Sidamon‐Eristoff AE , Gilson A , Chartash D . The role of large language models in medical education: applications and implications. JMIR Med Educ 2023; 9: e50945 37578830 10.2196/50945 PMC10463084

[19] Swoboda CM , Van Hulle JM , McAlearney AS , Huerta TR . Odds of talking to healthcare providers as the initial source of healthcare information: updated cross‐sectional results from the Health Information National Trends Survey (HINTS). BMC Fam Pract 2018; 19: 146 30157770 10.1186/s12875-018-0805-7 PMC6116497

[20] Cacciamani GE , Dell'Oglio P , Cocci A et al. Asking “Dr. Google” for a second opinion: the devil is in the details. Eur Urol Focus 2021; 7: 479–481 31690539 10.1016/j.euf.2019.10.011

[21] Rodler S , Kopliku R , Ulrich D et al. Patients' trust in artificial intelligence–based decision‐making for localized prostate cancer: results from a prospective trial. Eur Urol Focus 2024; 10: 654–661 37923632 10.1016/j.euf.2023.10.020

[22] Lennartz S , Dratsch T , Zopfs D et al. Use and control of artificial intelligence in patients across the medical workflow: single‐center questionnaire study of patient perspectives. J Med Internet Res 2021; 23: e24221 33595451 10.2196/24221 PMC7929746

[23] Carl N , Nguyen L , Haggenmüller S et al. Comparing Patient's confidence in clinical capabilities in urology: large language models versus urologists. Eur Urol Open Sci 2024; 70: 91–98 39507511 10.1016/j.euros.2024.10.009 PMC11538625

[24] Gallifant J , Afshar M , Ameen S et al. The TRIPOD‐LLM reporting guideline for studies using large language models. Nat Med 2025; 31: 60–69 39779929 10.1038/s41591-024-03425-5 PMC12104976

[25] Agresti A , Coull BA . Approximate is better than “exact” for interval estimation of binomial proportions. Am Stat 1998; 52: 119–126

[26] Rodler S , Maruccia S , Abreu A et al. Readability assessment of patient education materials on Uro‐oncological diseases using automated measures. Eur Urol Focus 2024. https://www.eu‐focus.europeanurology.com/article/S2405‐4569(24)00117‐2/fulltext 10.1016/j.euf.2024.06.012 39048402

[27] Zhu M , Patel RD , Dave P et al. Health‐related internet use among new urology clinic patients. Urology 2024; 194: 1–6. https://www.sciencedirect.com/science/article/pii/S0090429524008987 39395456 10.1016/j.urology.2024.10.023

[28] Longhurst CA , Singh K , Chopra A , Atreja A , Brownstein JS . A call for artificial intelligence implementation science centers to evaluate clinical effectiveness. NEJM AI 2024; 1: 8. 10.1056/AIp2400223

[29] Ouyang D , Hogan J . We need more randomized clinical trials of AI. NEJM AI 2024; 1: 11

[30] Huang H , Tang T , Zhang D et al. Not all languages are created equal in LLMs: Improving multilingual capability by cross‐lingual‐thought prompting [Internet]. *arXiv* 2023. http://arxiv.org/abs/2305.07004

