# Supplementary material for: Evaluating interactions of patients with large language models for medical information
Source: BJU Int. 2025 Feb 18;135(6):1010–7. doi: 10.1111/bju.16676 (PMC12053131; doi:10.1111/bju.16676)
Supplement: Supplementary file 2 — Table S1. Post‐interventional survey. Table S2. Demographic data and medical information‐seeking preferences. Table S3. Descriptive Table with the reason of admittance. Table S4. Results of a ordinal logistic regression model. Figure S1. Histogram detailing reason of admittance. [file BJU-135-1010-s001.docx]

Supplementary material for the manuscript: **Evaluating Interactions of Patients with Large Language Models for Medical Information**

**Supplementary Table 1: post-interventional survey**

| **Questions** | **Answers** |
| --- | --- |
| Have you ever used the internet (in the form of search engines like Google and others) for medical questions? | - Yes - No |
| How often do you use the internet for medical questions? | - Daily - Weekly - Monthly |
| Please specify which online sources you have used. | *free text answer* |
| **How much do you agree with the following statement?** | |
| How high is your confidence in the internet to find accurate medical information? | - Very high - Somewhat high - Neutral - Somewhat low - Very low |
| How high is your affinity for artificial intelligence technologies? | - Very high - Somewhat high - Neutral - Somewhat low - Not familiar at all |
| Have you heard of language models (for example ChatGPT, Gemini or other)? | - Yes - No |
| Have you ever used language models for medical questions? | - Yes - No |

**Supplementary Table 1: post-interventional survey**

| **Question** | **Antwort** |
| --- | --- |
| **How much do you agree with the following statement?** | |
| I enjoyed talking with the chatbot. | - Very high - Somewhat high - Neutral - Somewhat low - Very low |
| I had the feeling the chatbot understood my questions. | - Very high - Somewhat high - Neutral - Somewhat low - Very low |
| The chatbot made a competent impression on me. | - Very high - Somewhat high - Neutral - Somewhat low - Very low |
| The chatbot provided useful information. | - Very high - Somewhat high - Neutral - Somewhat low - Very low |
| The chatbot provided complete answers to my questions. | - Very high - Somewhat high - Neutral - Somewhat low - Very low |
| The chatbot explained the provided information sufficiently. | - Very high - Somewhat high - Neutral - Somewhat low - Very low |
| I understood the content of the provided information well. | - Very high - Somewhat high - Neutral - Somewhat low - Very low |
| I understood the technical language of the provided information well. | - Very high - Somewhat high - Neutral - Somewhat low - Very low |
| The chatbot was easy to use. | - Very high - Somewhat high - Neutral - Somewhat low - Very low |
| The language model was able to answer my questions better than a search engine. | - Very high - Somewhat high - Neutral - Somewhat low - Very low |
| I understood the language model better than the doctor. | - Very high - Somewhat high - Neutral - Somewhat low - Very low |
| The language model was able to answer my questions better than a doctor. | - Very high - Somewhat high - Neutral - Somewhat low - Very low |
| I would use a language model (for medical information) again. | - Very high - Somewhat high - Neutral - Somewhat low - Very low |

**Demographic data and medical information-seeking preferences**

As this study presents findings within the context of a larger, multifaceted research effort, we present demographic data and information-seeking preferences as supplementary material because they are already embedded in a separate manuscript associated with the IPALLM study [1].

To investigate information-seeking preferences among patients, we first assessed the preferred source of medical information, confidence in online sources and affinity for AI-technologies. This section presents the findings from the survey conducted in the pre-interventional phase. For detailed results please refer to **Supplementary Table 2.** The majority of the sample (239/292; 82%) used Google as primary source for medical information, with nearly half of them (139/292; 48%) using it daily. More participants (103/292; 35%) responded to have ‘rather low’ or ‘very low’ confidence in online sources for medical information compared to ‘rather high’ or ‘very high’ confidence (47/292; 15.7%). Approximately half of the sample (153/292; 53%) indicated a ‘rather low’ or ‘very low’ affinity for AI technologies, while less participants (56/292; 19%) reported a ‘rather high’ or ‘very high’ affinity. Notably, 89% (259/292) of participants have never used LLMs for medQA. A significant difference in LLM-usage for medQA was observed across age groups, with increased usage among ‘Young Adults’ (6/23; 26%) and ‘Adults’ (6/27; 22% p-value for age group difference <0.001). Together, this data shows that patients predominantly rely on Google in medical information-seeking and half exhibit low confidence in online sources. Also, patients reported limited affinity for AI technologies, with minimal prior exposure to LLMs especially in senior and elderly patients, which comprise the majority of urological patients.

**Supplementary Table 2: Demographic data and medical information-seeking preferences:** Separated by age groups including Young Adult (18-30), Adult (30-50), Senior (50-65) and Elderly (>65), age in years, educational levels according to the 2001 International Standard Classification of Education (ISCED), survey results (n=292) on information-seeking preferences, AI affinity and use of LLM for medical questions, medQA = medical Question-Answering, SD = standard deviation. For better readability, percentages in this table are rounded to whole numbers. Demographic data was collected on a voluntary basis, and any missing data was excluded from the analysis.

| **Characteristic** | **Overall**, N = 292*^1^* | **Young Adult**, n = 23*^1^* | **Adult**, n = 28*^1^* | **Senior**, n = 96*^1^* | **Elderly**, n = 145*^1^* |
| --- | --- | --- | --- | --- | --- |
| **Age (years)^2^** | 60.58 (15.7) | 23.61 (2.9) | 38.14 (5.6) | 58.73 (4.0) | 71.99 (5.4) |
| **Gender** |  |  |  |  |  |
| m | 212 (73%) | 11 (48%) | 16 (57%) | 71 (74%) | 114 (79%) |
| w | 71 (24%) | 12 (52%) | 11 (39%) | 24 (25%) | 24 (17%) |
| **Education** | |  |  |  |  |
| 1 (ISCED 0-1) | 3 (1%) | 0 (0%) | 2 (7%) | 0 (0%) | 1 (1%) |
| 2 (ISCED 2-3) | 83 (28%) | 8 (35%) | 9 (32%) | 30 (31%) | 36 (25%) |
| 3 (ISCED 4-6) | 43 (15%) | 8 (35%) | 5 (18%) | 13 (14%) | 17 (12%) |
| 4 (ISCED 7-8) | 84 (29%) | 2 (9%) | 8 (29%) | 26 (27%) | 48 (33%) |
| **Comorbidities** |  |  |  |  |  |
| Present | 151 (52%) | 1 (4%) | 10 (35%) | 52 (54%) | 88 (60%) |
| **Living arrangement** | |  |  |  |  |
| Alone | 44 (15%) | 3 (13%) | 10 (36%) | 15 (16%) | 16 (11%) |
| In Cohabitation | 187 (64%) | 14 (61%) | 13 (46%) | 63 (84%) | 97 (67%) |
| **Do you use Google (or other search engines) for medical information?** | | | | |  |
| Yes | 239 (82%) | 22 (96%) | 27 (96%) | 81 (84%) | 109 (75%) |
| No | 53 (18%) | 1 (4%) | 1 (4%) | 15 (16%) | 36 (25%) |
| **Frequency use for medical information** | | |  |  |  |
| Monthly | 53 (18%) | 2 (9%) | 1 (3.6%) | 16 (17%) | 34 (23%) |
| Weekly | 100 (34%) | 9 (39%) | 13 (46%) | 34 (35%) | 44 (30%) |
| Daily | 139 (48%) | 12 (52%) | 14 (50%) | 46 (48%) | 67 (46%) |
| **Confidence in online sources?** | |  |  |  |  |
| Very Low | 38 (13%) | 6 (26%) | 1 (4%) | 16 (17%) | 15 (10%) |
| Rather Low | 65 (22%) | 3 (13%) | 10 (36%) | 21 (22%) | 31 (21%) |
| Neutral | 142 (49%) | 11 (48%) | 12 (43%) | 49 (51%) | 70 (48%) |
| Rather High | 39 (13%) | 3 (13%) | 5 (18%) | 9 (9%) | 22 (15%) |
| Very High | 8 (3%) | 0 (0%) | 0 (0%) | 1 (1%) | 7 (5%) |
| **How high is your affinity for artificial intelligence technologies?** | | | |  |  |
| Very Low | 58 (20%) | 0 (0%) | 2 (7%) | 17 (18%) | 39 (27%) |
| Rather Low | 95 (33%) | 6 (26%) | 7 (25%) | 36 (38%) | 46 (32%) |
| Neutral | 83 (28%) | 5 (22%) | 7 (25%) | 28 (29%) | 43 (30%) |
| Rather High | 46 (16%) | 8 (35%) | 11 (39%) | 14 (15%) | 13 (9%) |
| Very High | 10 (3%) | 4 (17%) | 1 (4%) | 1 (1%) | 4 (3%) |
| **Have you ever heard of ChatGPT or other LLMs?** | | |  |  |  |
| Yes | 201 (69%) | 22 (96%) | 24 (86%) | 62 (65%) | 93 (64%) |
| No | 91 (31%) | 1 (4%) | 4 (14%) | 34 (35%) | 52 (36%) |
| **Have you ever used ChatGPT or other LLM for medical questions?** | | |  |  |  |
| Yes | 22 (8%) | 6 (26%) | 6 (21%) | 6 (6%) | 4 (2%) |
| No | 259 (89%) | 17 (74%) | 21 (75%) | 87 (90%) | 134 (92%) |
| *^1^* n / N (%); *^2^* Mean (SD) | | | | | |

**Supplementary Table 3: Descriptive Table with the reason of admittance**

| **Characteristic** | **N = 292***^1^* |
| --- | --- |
| Reason of admittance grouped by procedure |  |
| Prostate biopsy | 48 (16.2%) |
| BPH* | 30 (10.1%) |
| Kidney tumor surgery** | 25 (8.4%) |
| Testicular cancer surgery | 4 (1.4%) |
| Bladder cancer surgery*** | 39 (13.2%) |
| Urolithiasis**** | 26 (8.8%) |
| RAPN | 46 (15.5%) |
| Others****** | 52 (17.6%) |
| Unknown | 26 (8.8%) |
| *^1^* n (%) | |

‘BPH**’ refers to benign prostatic hyperplasia, including holmium laser enucleation of the prostate and prostatic artery embolization. ’Kidney tumor surgery**’ includes partial nephrectomies and nephrectomies. ’Bladder cancer surgery****’ includes transurethral resection of bladder tumors and radical cystectomies. ‘Urolithiasis****’ includes ureteroscopy, lithotripsy, and percutaneous nephrolithotripsy. ‘Other*****’ includes reconstructive and transgender surgery, as well as minor procedures like elective DJ stent changes or circumcisions.

**Supplementary Figure 1: Histogram detailing reason of admittance**

**
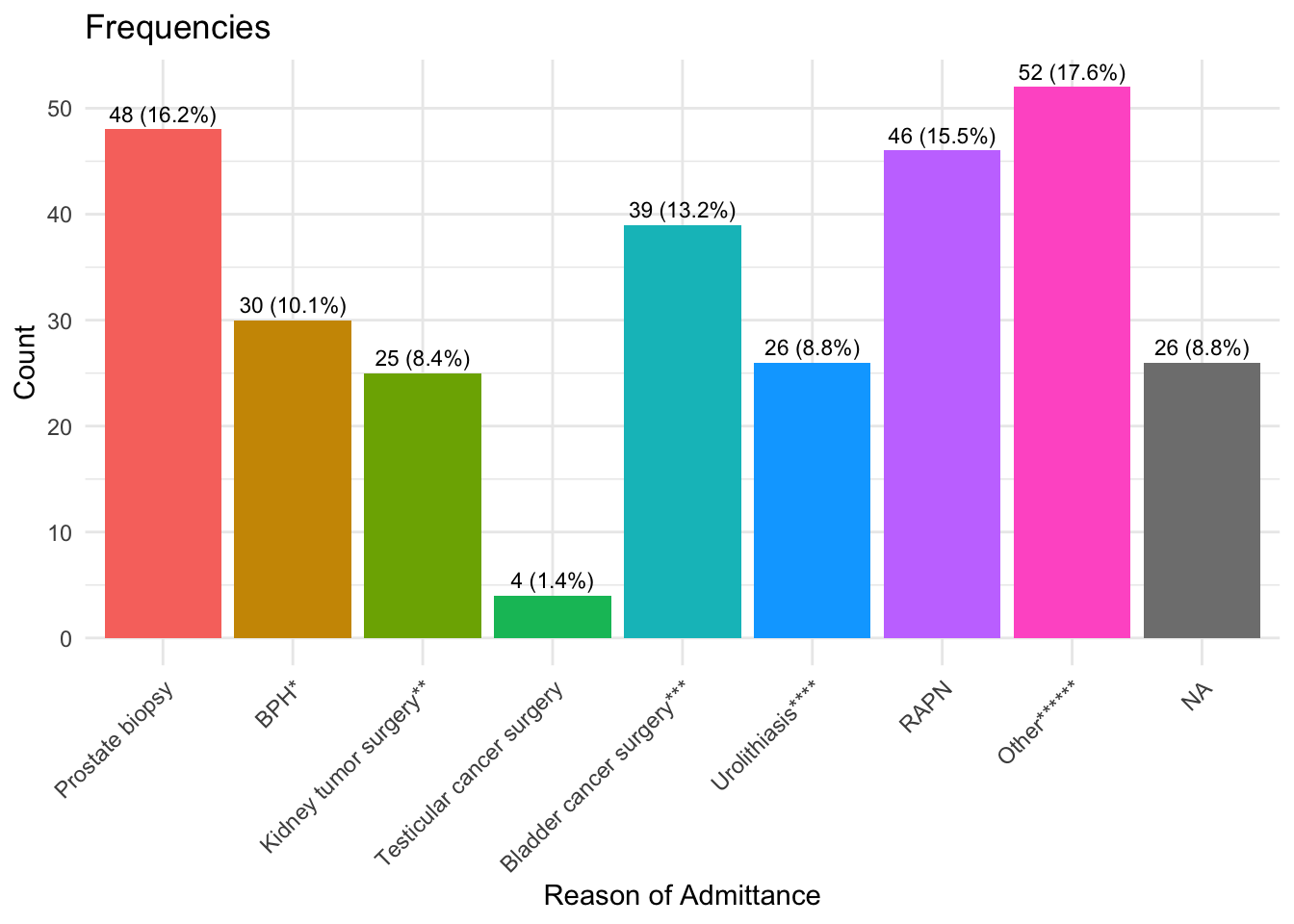
**

**Supplementary Figure 1:** The reason for admittance was collected along with demographic data at the end of the survey. Reasons for admittance were grouped into common urological subfields. NA (Not Applicable) refers to patients who did not want to disclose their reason for admittance.

‘BPH**’ refers to benign prostatic hyperplasia, including holmium laser enucleation of the prostate and prostatic artery embolization. ’Kidney tumor surgery**’ includes partial nephrectomies and nephrectomies. ’Bladder cancer surgery****’ includes transurethral resection of bladder tumors and radical cystectomies. ‘Urolithiasis****’ includes ureteroscopy, lithotripsy, and percutaneous nephrolithotripsy. ‘Other*****’ includes reconstructive and transgender surgery, as well as minor procedures like elective DJ stent changes or circumcisions.

**Supplementary Table 4:** Results of a ordinal logistic regression model.

| **Question** | **Factor/Coefficient** | **Odds Ratio** | **lower bound** | **upper bound** | **p value** |
| --- | --- | --- | --- | --- | --- |
| The chatbot was able to answer questions better than a search engine. | Age | 0.99 | 0.97 | 1.00 | 0.13 |
|  | Affinity for Artificial Intelligence | 0.94 | 0.74 | 1.21 | 0.64 |
|  | Used ChatGPT prior | 0.54 | 0.22 | 1.32 | 0.17 |
|  | Confidence in online sources | 0.84 | 0.65 | 1.08 | 0.17 |
|  | Education | 0.91 | 0.74 | 1.13 | 0.39 |
| I understood the chatbot better than a urologist. | Age | 0.99 | 0.97 | 1.01 | 0.18 |
|  | Affinity for Artificial Intelligence | 0.88 | 0.67 | 1.16 | 0.37 |
|  | Used ChatGPT prior | 0.56 | 0.20 | 1.54 | 0.26 |
|  | Confidence in online sources | **1.34** | **1.00** | **1.80** | **0.05** |
|  | Education | **0.73** | **0.56** | **0.93** | **0.013** |
| The chatbot was able to answer my questions better than a urologist. | Age | 1.01 | 0.99 | 1.03 | 0.28 |
|  | Affinity for Artificial Intelligence | **0.63** | **0.46** | **0.87** | **0.005** |
|  | Used ChatGPT prior | **0.38** | **0.15** | **1.00** | **0.049** |
|  | Confidence in online sources | 1.11 | 0.80 | 1.54 | 0.53 |
|  | Education | **0.60** | **0.44** | **0.80** | **0.001** |

[1] Carl N, Nguyen L, Haggenmüller S, Joachim Hetz M, Theres Winterstein J, Otto Hartung F, et al. Comparing Patient’s Confidence in Clinical Capabilities in Urology: Large Language Models Versus Urologists. Eur Urol Open Sci 2024;70:91–8. https://doi.org/10.1016/j.euros.2024.10.009.
